# Cremation of Dr. Colletti

**Published:** 1881-07

**Authors:** 


Cremation of Dr. Colletti.—We learn from an exchange that Dr. Ferdinand Colletti, Professor of therapeutics at the University of Padua, who died a short while since, was cremated at Milan, with distinguished honors.

Professor Colletti was one of the earlier champions and defenders of cremation, and directed that his remains should be disposed of in accordance with his favorite theory. We are glad to call attention to the manner in which the subject of cremation is making its way among thinking men of the times.

